# From fluid loss to calcification: MRI and CT matrix features in cystic echinococcosis

**DOI:** 10.1186/s13244-026-02375-5

**Published:** 2026-09-03

**Authors:** Marine Julien, Louis Bohard, Andréas Busse-Coté, Noémie Tissot, Celia Turco, Coralie Barerra, Laurence Millon, OFREKYS Group, Delphine Weil-Verhoeven, Alexandre Doussot, Carine Richou, Raphaël Anxionnat, Corinne Bonny, Carole Hartmann, Hannah Gondran, André Paugam, Éric Caumes, Stylianos Tzedakis, Marie Cortier, Anna Stanzelova, Aurélie Plessier, Elora Peulier-Maitre, Anne-Laure Mounayar, Edouard Girard, Julie Arata-Bardet, Marie-Charlotte Chopin, Sophie Panaget, Fanny Vuotto, Emilia Frentiu, Élise Fiaux, Natacha Mrozek, Alina Tone, Nicolas Ettahar, Simona Tirziu, Cassandra Rayer, Baptiste Giguet, Hikombo Hitoto, Jean-Baptiste Laine, Marc Decrock, Vincent Gendrin, Lucas Perez, Sophie Mahy, Baptiste Hoellinger, Pierre Toumelin, Émilie Piet, Nolwenn Laborde, Jean-François Faucher, Roxana Genet, Margaux Miralles, Tayma Naciri, Théo Matejka, Caroline Zhang, Marine Morrier, Amélie Dureault, Nicolas Flori, Jean-Christophe Bendini, Solange Bresson-Hadni, Paul Calame

**Affiliations:** 1 Department of Radiology, University Marie and Louis Pasteur and University Hospital Besançon, Besançon, France; 2 Department of Infectious Disease, University Marie and Louis Pasteur and University Hospital Besançon, Besançon, France; 3 Department of Radiology, Hôpital Beaujon, AP-HP.Nord, Clichy, France; 4 Department of Digestive Surgery, University Marie and Louis Pasteur and University Hospital Besançon, Besançon, France; 5 OFREKYS Group, Besançon, France; 6 National Reference Center for Echinococcoses, Department of Parasitology-Mycology, University Hospital Besançon, Besançon, France; 7 University Marie and Louis Pasteur, CNRS, Chrono-environment Research Unit, Besançon, France; 8 Université Paris Cité, INSERM Centre de Recherche sur l’Inflammation, Paris, France; 9 University Hospital Besançon, Besançon, France; 10 University Hospital Nantes, Nantes, France; 11 AP-HP, Paris, France; 12 University Hospital Grenoble, Grenoble, France; 13 University Hospital Lille, Lille, France; 14 University Hospital Nancy, Nancy, France; 15 University Hospital Rouen, Rouen, France; 16 University Hospital Clermont-Ferrand, Clermont, France; 17 Valenciennes General Hospital, Valenciennes, France; 18 Métropole Savoie General Hospital, Chambéry, France; 19 University Hospital Rennes, Rennes, France; 20 Le Mans General Hospital, Le Mans, France; 21 Hôpital Nord Franche-Comté, Trévenans, France; 22 University Hospital Montpellier, Montpellier, France; 23 University Hospital Dijon, Dijon, France; 24 University Hospital Strasbourg, Strasbourg, France; 25 Roubaix General Hospital, Roubaix, France; 26 Annecy Genevois General Hospital, Epagny Metz-Tessy, France; 27 University Hospital Toulouse, Toulouse, France; 28 University of Limoges, Limoges, France; 29 Metz-Thionville Regional Hospital Center, Thionville, France; 30 University Hospital Nîmes, Nîmes, France; 31 GHICL, Lille Catholic Hospitals, Lille, France; 32 GHT 85, Vendée, Pays de la Loire region, France; 33 Valence General Hospital, Valence, France; 34 ICM–Montpellier Cancer Institute, Montpellier, France; 35 Agahtir, Nice, France

**Keywords:** Echinococcosis, Magnetic resonance imaging, Tomography, X-ray computed, Diagnosis, differential, Cysts

## Abstract

**Objective:**

To characterize MRI matrix signal and CT calcification patterns across WHO-IWGE stages, compare them with lesion mimicking cystic echinococcosis (CE), and derive an MRI-based subclassification for inactive cysts.

**Materials and methods:**

This retrospective multicenter study included 105 patients (174 CE cysts) imaged between 2012 and 2024, plus 30 mimickers. Inclusion required axial T1-weighted imaging with chemical-shift in-phase/opposed-phase acquisitions and at least two T2-weighted sequences. Two radiologists independently reviewed all examinations, and a consensus was reached. Cysts were classified as inactive if no free-fluid T2 hypersignal was present on index MRI and corroborated by follow-up imaging and/or adjunct ultrasound in equivocal cases. Diagnostic performance indices were calculated to identify inactivity.

**Results:**

Fatty T1 hyperintensity was more frequent in inactive than active/transitional cysts (20/97 (21%) vs 2/77 (3%); *p* < 0.001). The “matrix flip-flop” sign was observed in 61/97 (63%) inactive cysts and in only two CE3b lesions (2/77 (3%); *p* < 0.001). Both signs showed high specificity for inactivity (75/77; 97.4%, 95% CI: 90.9–99.7). Matrix calcifications were associated with inactivity (46/97 (47%) vs 2/77 (3%); *p* < 0.001) and were rare among mimickers (2/30 (7%); *p* = 0.006). Homogeneous non-fatty T1 hyperintensity characterized mimickers (23/30 (77%) vs 10/174 (6%); *p* < 0.001). A three-tier inactive subclassification (early/intermediate/late) was derived from matrix T2 evolution.

**Conclusion:**

Matrix MRI/CT features—particularly fatty foci and the matrix flip-flop sign—provide ancillary evidence of CE inactivity and help distinguish CE from mimickers. T2 signal supported an MRI-based framework for stage-adapted “watch-and-wait” follow-up.

**Key Points:**

- **Question** MRI features of cystic echinococcosis remain insufficiently studied, particularly matrix signal abnormalities that may affect diagnosis and viability assessment on routine imaging.
- **Findings** T1- and T2-weighted matrix CE cysts analysis identified fatty foci and the newly described matrix flip-flop sign as specific features of inactive cysts.
- **Critical Relevance** Assessment of underrecognized MRI and CT cyst-matrix features can help stage cystic echinococcosis, assess viability, and distinguish cystic echinococcosis cysts from mimickers.

**Graphical Abstract:**

**Figure:**
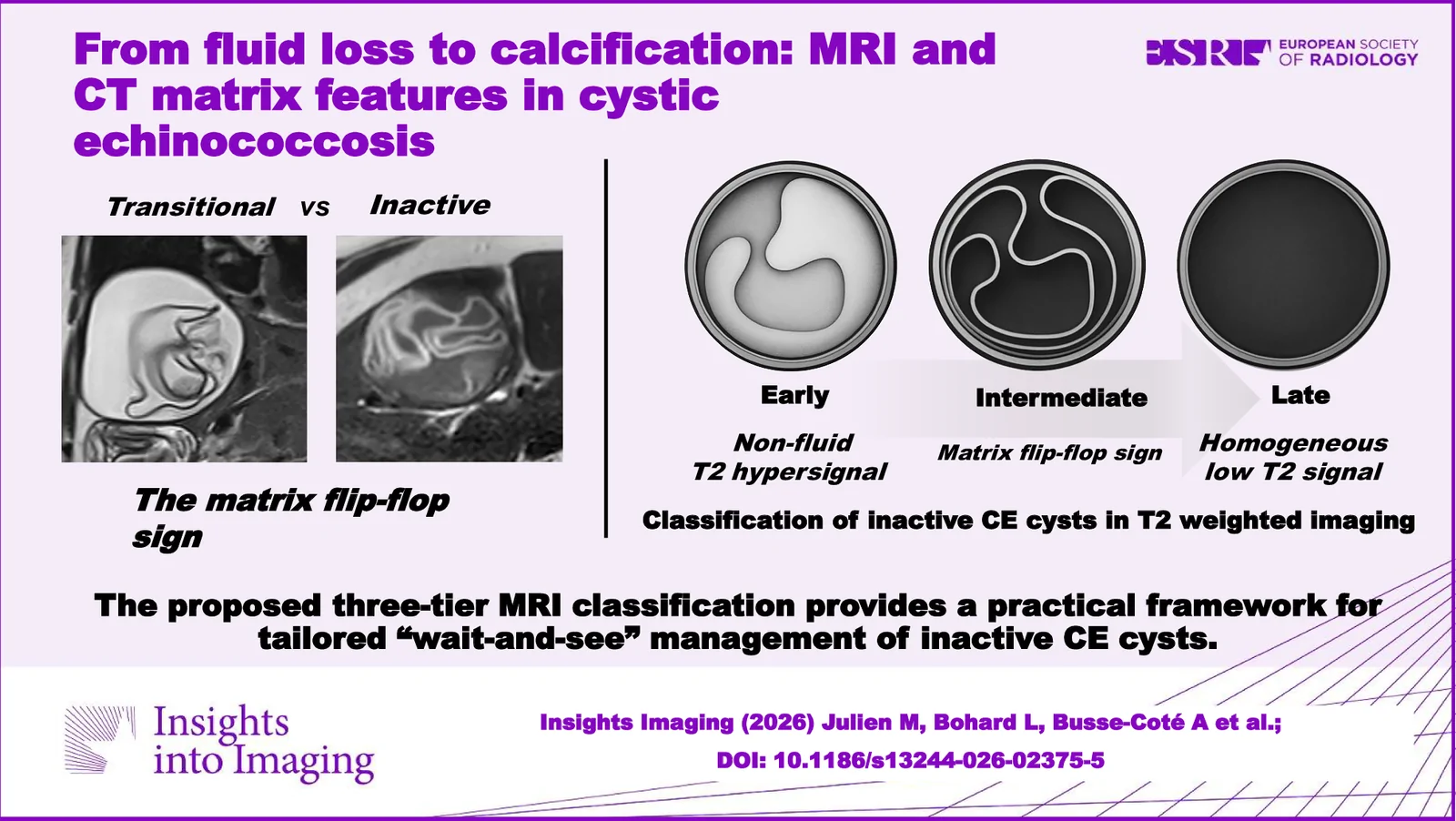

## Introduction

Cystic echinococcosis (CE) is a zoonosis produced by the larval stage of *Echinococcus granulosus*. It remains endemic in large parts of Africa, Australia, the Middle East, and South America; France is a low-endemic zone, and the majority of cases are imported except in Corsica and the South-East area of France [1, 2]. CE is slow-growing and largely silent, with 70% of cysts in the liver, 20% in the lungs, and the remainder scattered among spleen, kidney, muscle, bone, heart, and brain [3].

Serology alone is insufficiently sensitive for diagnosis but also for monitoring, so imaging underpins diagnosis and management [4]. Ultrasound (US) is inexpensive, widely available, and, in endemic areas, forms the basis of the World Health Organization–Informal Working Group on Echinococcosis (WHO-IWGE) staging system, which classifies cysts as active (CE1-2), transitional (CE3a and CE3b), or inactive (CE4-5) [3, 5–7]. US reliably detects intracystic fluid and daughter cysts, but its performance drops when the wall is densely calcified. Moreover, it is far less effective outside the liver [8].

Computed tomography (CT) and magnetic resonance imaging (MRI) are now frequently used for incidental detection, especially in industrialized countries. CT is a highly accurate modality for visualizing calcifications and complications. However, its efficacy is suboptimal for the analysis of cyst contents and staging [6]. MRI—particularly heavily T2-weighted sequences—is comparable to US for fluid content, daughter cysts, and detached internal membranes, and an MRI-adapted WHO-IWGE scale correlates well with the original US classification [6, 7].

Yet, the distinction between active and inactive disease on MRI often hinges on the detection of a millimeter-thin fluid rim, whereas the matrix signal on T1-weighted images and calcification patterns has been only sparsely investigated. Previous reports link intralesional fat to occult biliary communication [9] or to degeneration [10, 11]. In addition, evidence concerning the distribution of calcium within the external wall, matrix, or detached metacestode membranes is limited, and a recent study reported no association between the degree of calcification and cyst activity [8]. Importantly, the broad “inactive” category (CE4–CE5) includes both cysts just beginning matrix mineralization and those that are already densely and irreversibly calcified, conditions that most likely entail distinct prognoses and follow-up requirements. Moreover, prior comparative work [6] assessing the transposition of WHO-IWGE staging from ultrasound to cross-sectional imaging reported high agreement for CE1–CE3 with MRI but only moderate agreement for CE4 and CE5.

Our primary objective was therefore to characterize MRI matrix signal and CT calcification patterns across WHO-IWGE stages and to compare them with cystic echinococcosis mimickers (MCE) lesions. Our secondary objective was to refine the classification of inactive cysts by stratifying CE lesions based on their T2-weighted matrix appearance, aiming to provide a coherent framework for stage-adapted “watch-and-wait” follow-up.

## Materials and methods

### Patients

This retrospective, multicenter study was conducted at the National Reference Center on Echinococcoses (NRC-E, University Hospital of Besançon). Between January 2012 and May 2024, all patients diagnosed with CE who were referred to the national multidisciplinary team (MDT) consultation coordinated by the National Reference Center on Echinococcoses (NRC-E, University Hospital of Besançon) were assessed for eligibility. The sole inclusion criterion was the availability of MRI as part of the diagnostic workup. The clinical data were collected from the OFREKYS database, which is compliant with the European legal context of the General Data Protection Regulation (approval by the ‘Comité de Protection des Personnes’) and with the French regulation (CNIL declaration No: 903306 v1). Written informed consent was collected from the patients before data entry. All data in the database were pseudonymized. The study was conducted in accordance with the Declaration of Helsinki.

**Fig. 1:**
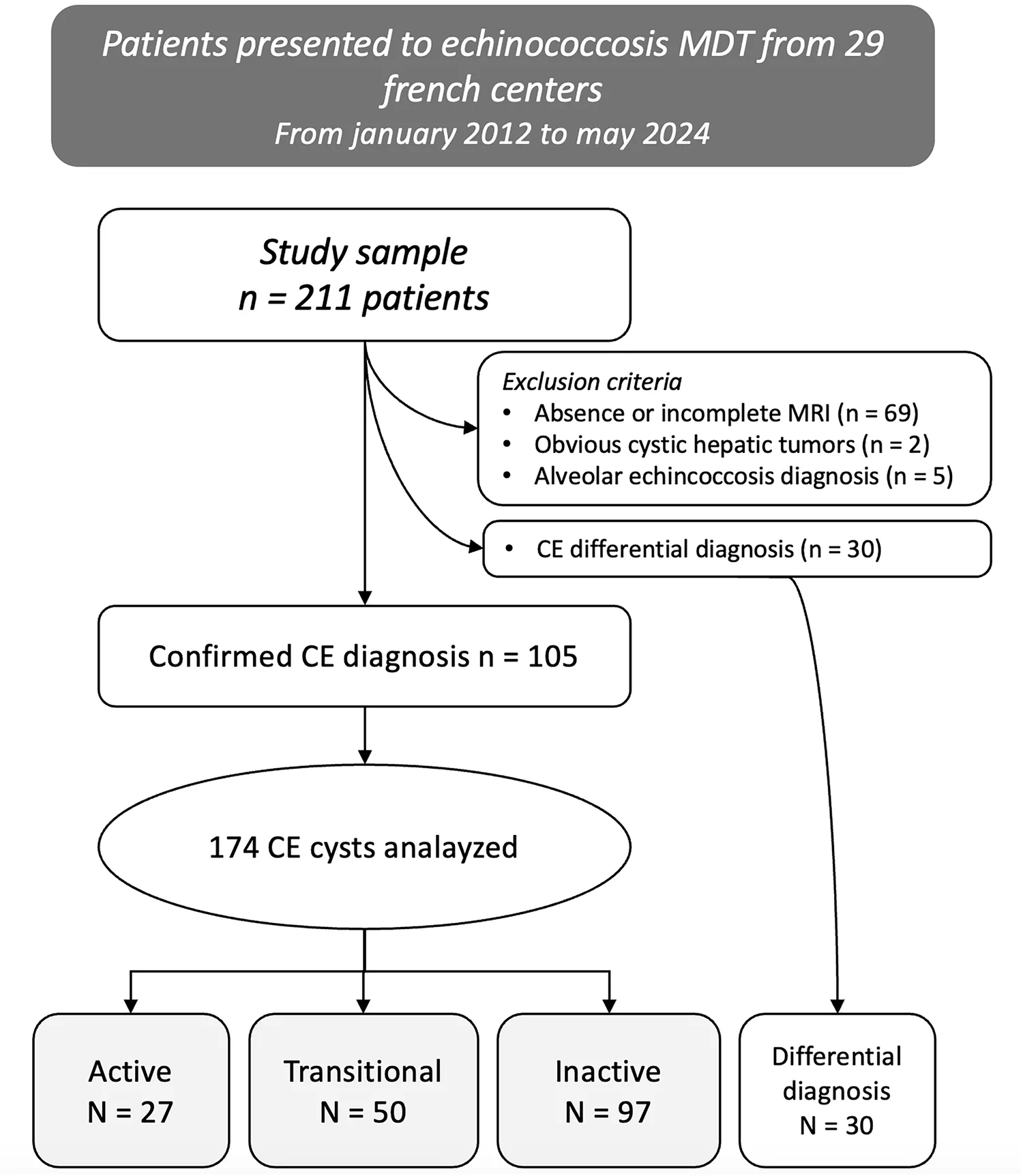
Flowchart of the study population

From an initial cohort of 211 patients, 69 were excluded due to absence or incomplete imaging. 5 patients were excluded following a definitive diagnosis of alveolar echinococcosis and 2 for an obvious liver cystic tumor lesion (Fig. 1).

For each included CE patient, clinical data, including sex, age, and significant past medical history, were collected. Treatment data related to CE, such as medical therapy with albendazole or surgical interventions (including type and date of surgery), were also recorded.

A separate comparison group of 30 patients with non-parasitic cystic lesions was also established. Patients were eligible for this group when they had a cystic lesion in any organ and had been referred to the multidisciplinary team because cystic echinococcosis was considered in the differential diagnosis. For this group, all patients with a cystic lesion were considered for inclusion, and patients were referred to the MDT for cystic echinococcosis suspicion. Obvious alternative diagnoses were excluded from this comparison group, including enhancing solid or infiltrative lesions. Alveolar echinococcosis was also excluded, as it typically presents as an infiltrative pseudo-tumoral lesion rather than as a purely cystic lesion. Cystic echinococcosis was ruled out when no imaging features suggestive of CE were present, including the absence of daughter cysts, detached membranes, or parasitic debris within the cyst. In equivocal cases, particularly for CE1 cysts mimicking simple cystic lesions, imaging follow-up was reviewed to avoid misclassification of early CE cysts as non-parasitic cysts.

In this MCE group, there were 16/30 (53.3%) patients with hepatic hemorrhagic biliary cyst, 5/30 (16.7%) patients with splenic epidermoid cyst, 2/30 (6.7%) patients with hepatic ciliated-lined cyst, 2/30 (6.7%) patients with renal hemorrhagic cyst, 2/30 (6.7%) patients with splenic cystic lymphangioma, 2/30 (6.7%) patients with hepatic cystadenoma and 1/30 (3.3%) patient with splenic pseudocyst.

### Imaging techniques

MRI was performed on 1.5-T and 3-T systems using institutional protocols; therefore, detailed sequence parameters were not fully standardized. However, all examinations met minimum requirements, and the following acquisitions (or their vendor-equivalent) were required for inclusion.

T2-weighted imaging. Examinations had to include at least two T2-weighted sequences: (1) a heavily T2-weighted single-shot fast spin-echo acquisition (HASTE/SSFSE/single-shot FSE-type) obtained in the axial and/or coronal plane in breath-hold (TR: very long, sequence-dependent; effective TE: 800–1200 ms), and (2) an axial T2-weighted fat-suppressed fast/turbo spin-echo sequence acquired with respiratory triggering/synchronization (or free breathing when triggering was not available) using a long TR and intermediate-to-long TE (TR: 2000–6000 ms; TE: 70–110 ms).

T1-weighted imaging. Axial T1-weighted imaging without and with fat suppression was obtained using gradient-echo or spin-echo techniques, most commonly in breath-hold (TR: 3–200 ms, depending on GRE vs SE; TE: 1–5 ms for GRE, or 8–15 ms for SE). Chemical-shift imaging with in-phase and opposed-phase acquisitions was systematically performed and was required for inclusion, acquired in breath-hold (typical TE: 1.1/2.2 ms at 1.5 T and 0.9/1.8 ms at 3 T; vendor-dependent).

MRCP, when performed for suspected biliary communication, consisted of heavily T2-weighted single-shot fast spin-echo sequences and/or a 3D heavily T2-weighted acquisition, acquired in breath-hold or with respiratory triggering depending on sequence and patient cooperation [12]. CT scans, when available, including at least an unenhanced acquisition phase, were reviewed.

Diffusion weighted imaging and post-contrast T1-weighted sequences were available in most examinations as part of routine MRI protocols, but they were not required for inclusion and were not evaluated for the present study. CE staging and activity assessment were based on T1/T2 matrix features, whereas contrast-enhanced imaging was used only to exclude obvious enhancing solid or infiltrative lesions.

### Imaging analysis

Cyst location (categorized as liver, lung, spleen, kidney, bone and muscle, or intraperitoneal) was initially recorded, followed by measurement of cyst size (the largest diameter was noted).

**Fig. 2:**
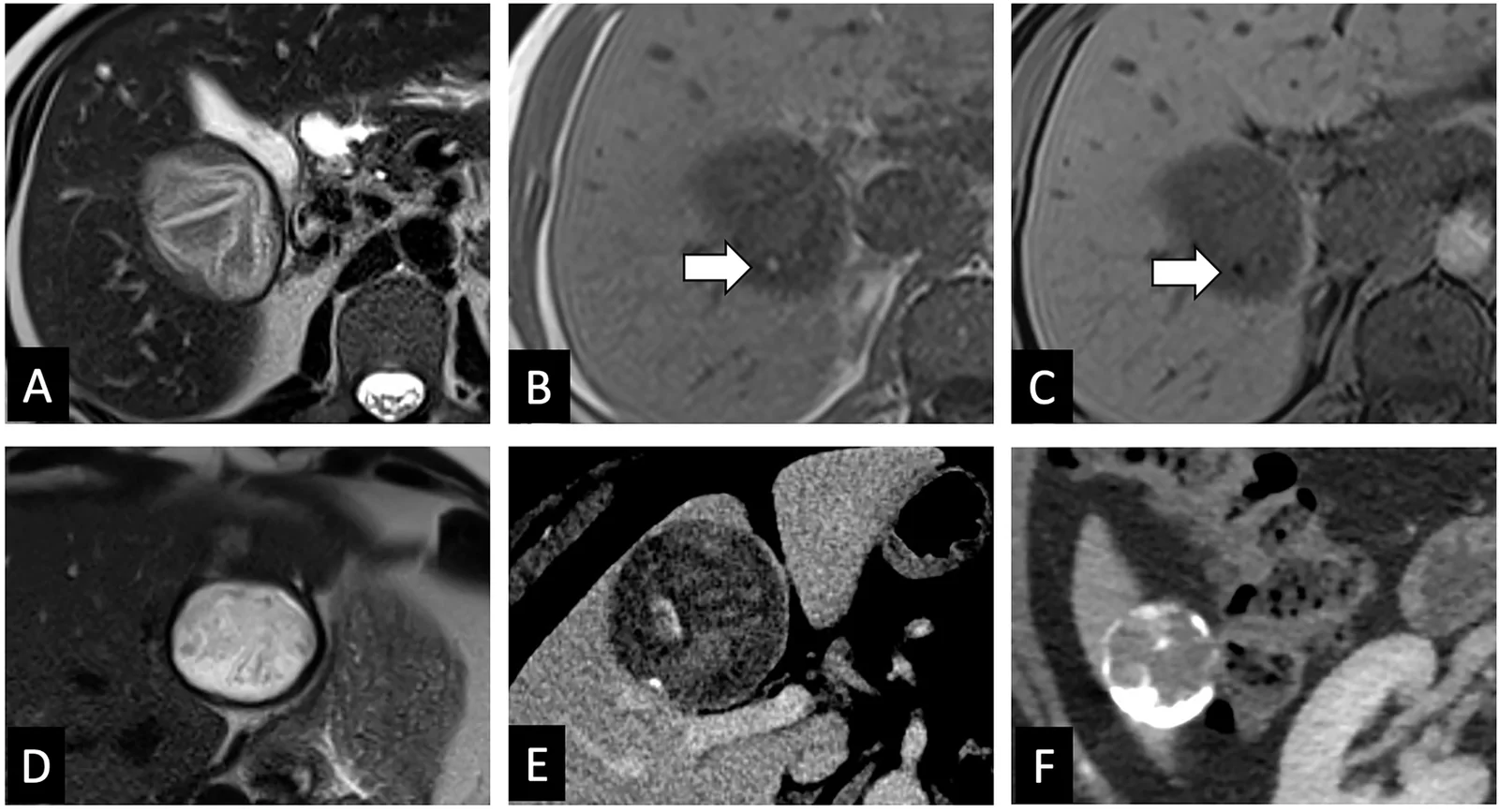
Example of main MRI features associated with parasitic activity. **A** Matrix flip-flop sign in T2-weighted imaging showing an inverted signal of internal membranes in hypersignal and matrix in hyposignal. **B** Presence of fatty hypersignal spot within the CE cyst in T1-weighted imaging in phase, in hypersignal (arrow). **C** Presence of fatty hypersignal spot within the CE cyst in T1-weighted imaging in opposed-phase, in hyposignal (arrow). **D** Inactive cystic echinococcosis lesion constituted by non-fluid hypersignal T2-weighted. **E** Cystic echinococcosis cyst with matrix calcifications. **F** Cystic echinococcosis cyst with internal membranes calcifications

Subsequently, MRI features of the cyst matrix were retrospectively evaluated as follows:

(i) Fatty T1 hypersignal: defined by the presence of macroscopic fat within the matrix, identified as an area of high signal intensity on T1-weighted in-phase images with a corresponding signal decrease on T1-weighted opposed-phase images (chemical shift artifact), typically appearing hyperintense relative to adjacent reference tissue on in-phase sequences (Fig. 2) [12]. (ii) Non-fatty T1 hypersignal: defined as a signal intensity of the matrix on T1-weighted images that was noticeably higher than that of the adjacent reference tissue, in the absence of findings for fatty T1 hypersignal (Fig. 2). (iii) Fluid T2 hypersignal: characterized by a markedly high signal intensity of the matrix on T2-weighted images, equivalent to that of simple fluids (e.g., cerebrospinal fluid or bile) and typically much higher than adjacent reference tissue. (iv) Non-fluid T2 hypersignal: defined as a signal intensity of the matrix on T2-weighted images that was higher than adjacent reference tissue but distinctly less intense than that of simple fluids (e.g., cerebrospinal fluid or bile), suggesting more complex or viscous content. (v) Isosignal (T1 or T2): defined as a signal intensity of the matrix on T1-weighted or T2-weighted images equivalent to that of the adjacent reference tissue. (vi) Hyposignal (T1 or T2): defined as a signal intensity of the matrix on T1-weighted or T2-weighted images noticeably lower than that of the adjacent reference tissue.

**Fig. 3:**
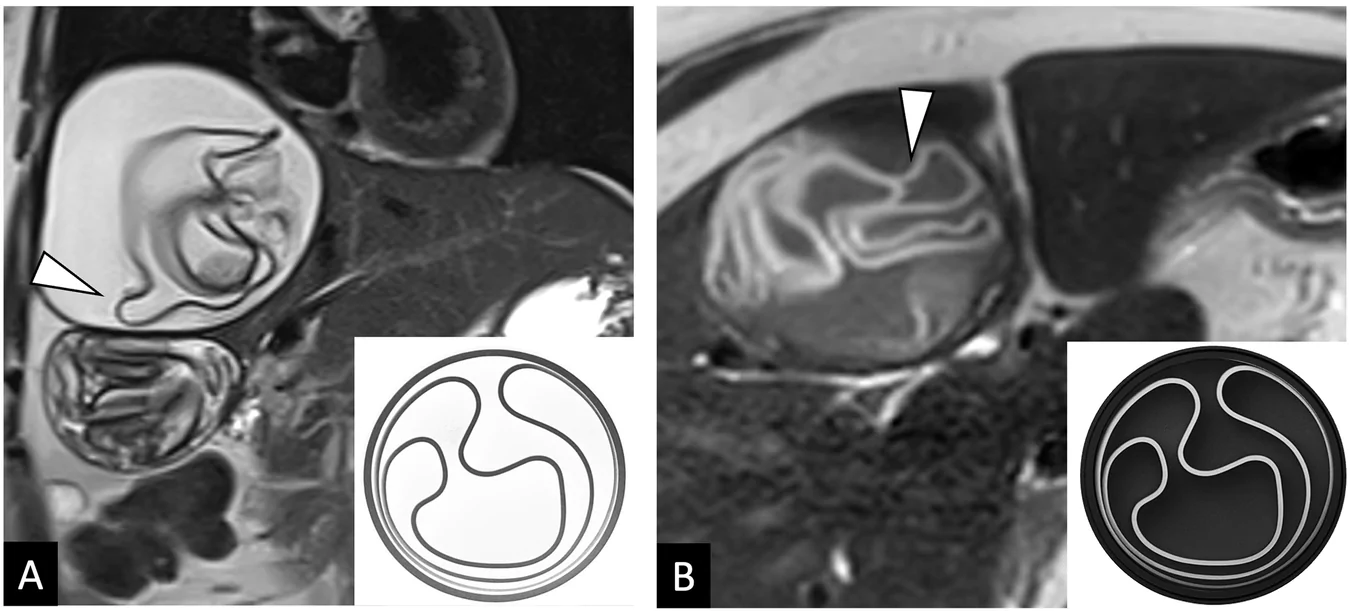
Illustration of the flip-flop matrix sign on MRI T2-weighted imaging. **A** Transitional cystic echinococcosis liver cyst constituted by liquid hypersignal in T2 associated with a detached germinal layer in low T2 signal (arrowhead), classified as CE3a according to the WHO-IWGE classification. **B** Inactive cyst characterized by a detached germinal layer in high-T2 signal (arrowhead) associated with a cyst matrix in low T2 signal, defining the specific flip-flop matrix sign

Additional morphological and signal characteristics on MRI were evaluated: (i) cyst contour features (regular, presence of outgrowths, fissuration, or biliary communication), (ii) the visibility of the parasitic membranes, and (iii) the presence of the MRI matrix signal inversion sign was recorded. The “flip-flop” matrix sign was defined as a T2 hyposignal of the cyst matrix contrasting with a T2 hypersignal of the detached parasitic membranes (Fig. 3).

The presence, number, and size of daughter cysts (identified as internal cystic structures exhibiting fluid T2 hypersignal within the parent CE cyst) were also documented.

CE cyst calcifications, assessed on available CT images, were categorized as: (i) Cyst wall calcification: defined by focal areas of high attenuation (150–1000 Hounsfield Units (HU)) within the cyst wall. (ii) Matrix calcification: defined by focal areas of high attenuation (150–1000 HU) within the cyst matrix. (iii) Internal membranes calcification: defined by focal areas of high attenuation (150–1000 HU) along the internal (parasitic) membranes.

Cysts were classified into six stages according to the WHO-IWGE system [13], adapted from ultrasound to MRI, as prior evidence indicates that heavily T2-weighted MRI reliably reproduces ultrasound-defined imaging features and staging in CE [6]: CE1: unilocular cyst with uniform fluid content. CE2: multivesicular cyst containing multiple daughter cysts within the parent cyst, with a fluid matrix. CE3a: cyst with detachment of the parasitic membranes (water-lily sign), with predominantly fluid content. CE3b: cyst with daughter cysts within a predominantly solid, degenerating matrix. CE4: cyst with heterogeneous, predominantly solid content without daughter cysts (e.g., “ball of wool” sign). CE5: solid-appearing cyst characterized by extensive calcification and a very low T2 signal.

### Cyst activity assessment and imaging review

In this study, cysts were operationally defined as ‘inactive’ if their MRI characteristics on the index examination showed no evidence of free fluid (i.e., absence of fluid T2 hypersignal, potentially presenting only with non-fluid T2 hypersignal). This MRI-based classification of inactivity was subsequently corroborated by either: (a) follow-up imaging demonstrating lesion stability, defined as no significant morphological changes and no new appearance of activity features (such as fluid T2 hypersignal or daughter cysts), and (b) adjunctive ultrasound findings supporting inactivity in cases where initial MRI findings were equivocal. Conversely, cysts were categorized as ‘active/transitional’ if any component exhibited fluid T2 hypersignal on the index MRI.

**Fig. 4:**
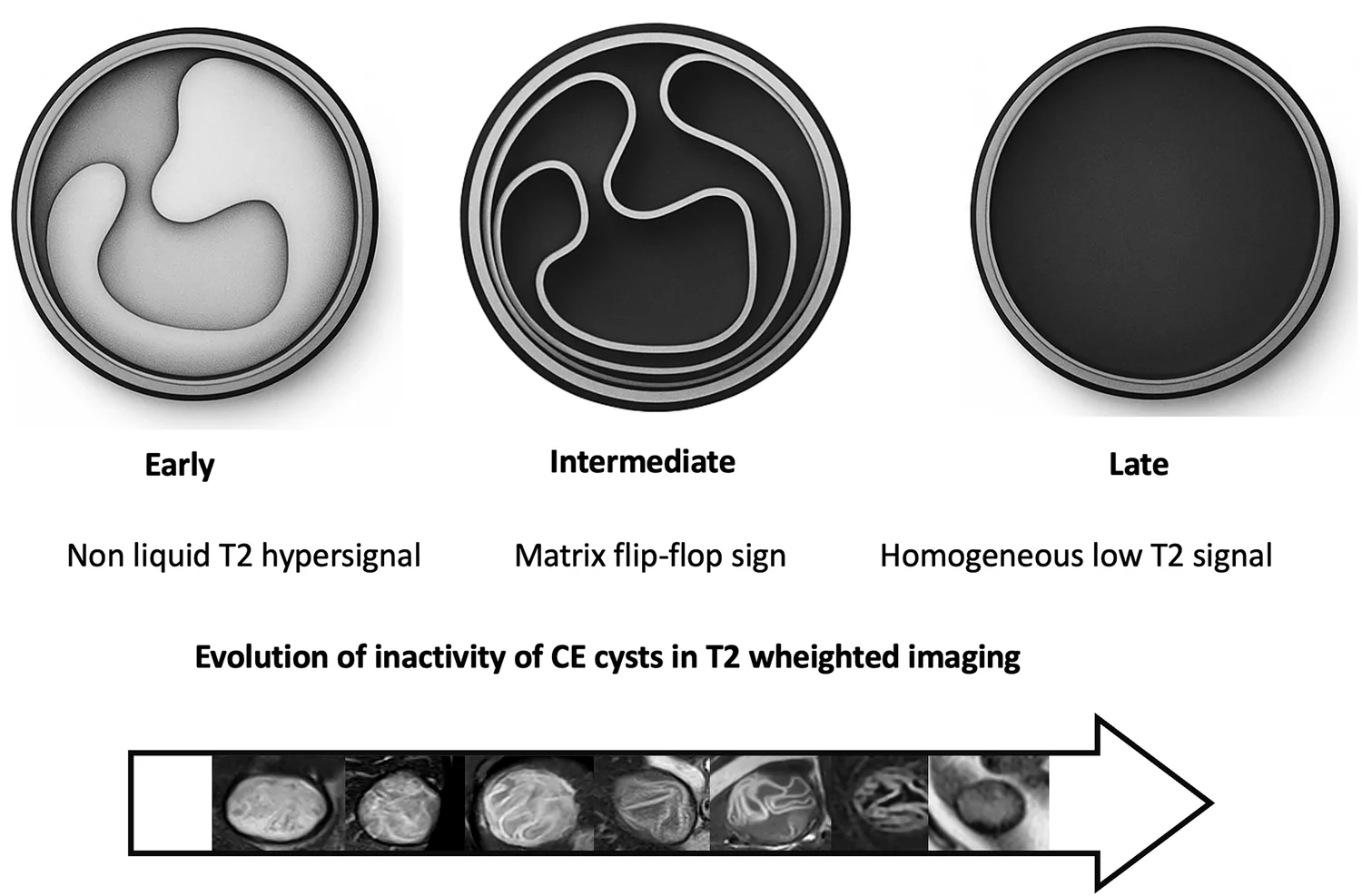
MRI-based three-tier subclassification of inactive CE cysts. Schematic illustration of the progressive transformation of the cyst matrix on T2-weighted imaging (arrow, left → right). Early-inactive stage: intracystic fluid signal has vanished, yet the matrix remains homogeneously hyperintense (“non-fluid” T2 hypersignal), reflecting the onset of laminated layer mineralization. Intermediate-inactive stage: the newly described “matrix flip-flop” sign emerges—defined as a T2 hyposignal of the cyst matrix contrasting with a T2 hypersignal of the detached parasitic membranes—signifying early matrix calcification. Late-inactive stage: uniformly hypointense matrix on both T1- and T2-weighted images, consistent with dense, confluent mineralization and debris compaction

Concerning inactive cysts, a new classification—Early-, intermediate-, and late-stage cysts—was defined by combining the WHO-IWGE stage with the T2-weighted inversion sign (Fig. 4):

- an early inactive cyst corresponded to a CE4 lesion with a high signal in T2-weighted imaging and without matrix T2 flip-flop sign inversion
- an Intermediate-inactive cyst to any CE4–CE5 lesion showing T2 matrix flip-flop sign T2
- a late-inactive cyst to a CE5 lesion without T2 inversion in a homogeneous low signal

All imaging data were retrospectively reviewed on a Picture Archiving and Communication System (PACS) workstation (Carestream Health Inc.; 2014 version) by two radiologists (M.J. and A.B.) with 4 and 6 years of experience in abdominal imaging, respectively. Any discrepancies in interpretation were resolved by consensus following a joint review with a third radiologist (P.C.) with 13 years of experience in abdominal imaging.

### Statistical analysis

Categorical data were reported as counts and percentages (*n*, %) and compared using the chi-squared (χ^2^) test or Fisher’s exact test, as appropriate. Continuous variables were assessed for normal distribution. Normally distributed data were expressed as mean ± standard deviation (SD) and compared using Student’s *t*-test. Non-normally distributed data were expressed as median and interquartile range (IQR) and compared using the Wilcoxon rank-sum test (Mann–Whitney U test).

To evaluate differences in imaging features of CE cysts based on their location (hepatic vs extrahepatic), initial univariate comparisons were performed. Subsequently, to account for potential confounding due to variations in WHO-IWGE stage distribution between locations, *p*-values were adjusted for WHO-IWGE classification.

Inter-reader agreement for the assessment of internal cyst matrix signal characteristics on MRI between the two primary radiologists was evaluated using the weighted kappa (κ) statistic and by the intraclass correlation coefficient (ICC) for the WHO-IWGE stage. Agreement levels were interpreted as: slight (κ = 0.00–0.20), fair (κ = 0.21–0.40), moderate (κ = 0.41–0.60), substantial (κ = 0.61–0.80), and almost perfect (κ = 0.81–1.00).

All statistical tests were two-sided, and a *p*-value < 0.05 was considered statistically significant. Analyses were conducted using RStudio (version 2013.12.1+402).

## Results

### Study population characteristics

The study cohort included 105 patients, with 58/105 men (55%) and 47/105 women (45%), with a median age of 44 years (IQR: 36; 56). A solitary lesion was present in 67/105 patients (64%), while 38/105 (36%) had multiple lesions, resulting in a total of 174 cysts being analyzed. The overall characteristics are detailed in Table 1.

**Table 1:** Characteristics of the study population

Cyst	*n* = 174
Location	
Liver, *n* (%)	145 (83)
Lung, *n* (%)	12 (7)
Spleen, *n* (%)	4 (2)
Bone and muscle, *n* (%)	5 (3)
Pelvis, *n* (%)	2 (1)
Retroperitoneal, *n* (%)	6 (4)
Pulmonary location (lobe)	
Right lower lobe, *n* (%)	6 (50)
Left lower lobe, *n* (%)	2 (17)
Right upper lobe, *n* (%)	2 (17)
Left upper lobe, *n* (%)	2 (16)
Size of cyst (cm)	7 [4; 11] (1.1–24)
Cyst with daughter cyst, *n* (%)	46 (26)
Daughter cyst size (cm)	2.5 [1; 4.2] (1–7.2)
Cyst morphology	
Outgrowth, *n* (%)	34 (20)
Biliary or pulmonary communication, *n* (%)	6 (3.4)
Fissuration	19 (11)
WHO-IWGE classification	
CE1	17 (10)
CE2	10 (6)
CE3a	12 (7)
CE3b	38 (22)
CE4	75 (43)
CE5	22 (13)
Medical management (105 patients)	
Medical treatment (albendazole), *n* (%)	79 (75)
Surgical treatment, *n* (%)	42 (40)
Undetermined, *n* (%)	4 (3.8)
Type of surgery	
Hepatic lobectomy, *n* (%)	5 (12)
Peri-cystectomy, *n* (%)	12 (28)
Subtotal hepatectomy, *n* (%)	11 (26)
Undetermined (%)	14 (33)

Numbers in parentheses are percentages

Of the 174 cysts, 145/174 (83%) were identified within the liver, while 29/174 (17%) were found in extrahepatic sites. Among the 29 extrahepatic cysts, 12/29 (41%) were in the lungs, 6/29 (21%) in the retroperitoneal space, 5/29 (17%) in bone and muscle, 4/29 (14%) in the spleen, and 2/29 (7%) in the pelvis. Within the liver, there was a predominance of only right lobe involvement (71/145 cysts; 49%) over the left lobe (41/145 cysts; 28%); the remaining cysts involved both lobes (33/145).

The overall median cyst size was 7.0 cm (IQR: 4.0; 11.0) (range: 1.1–24.0 cm). Morphological alterations included cyst outgrowth, observed in 34/174 cysts (20%), and fissuration in 19/174 cysts (11%). Communication with biliary or pulmonary structures was reported in 6/174 cysts (3.4%). Daughter cysts were present in 46/174 cases (26%), with a median size of 2.5 cm (IQR: 1.0; 4.2) (range: 1.0–7.2 cm).

Based on the WHO-IWGE classification, cysts were categorized into three activity groups for analysis: 27/174 (16%) were classified as active (CE1–CE2), 50/174 (29%) as transitional (CE3a–CE3b), and 97/174 (56%) as inactive (CE4–CE5). Median follow-up of inactive CE cysts was 60 months (IQR: 48–84).

### Inter-reader agreement of imaging features

Inter-reader agreement was moderate for fatty T1 hyperintensity (κ = 0.50 [95% CI: 0.28; 0.70]) and for non-fatty T1 hyperintensity (κ = 0.65 [95% CI: 0.45; 0.77]). In contrast, agreement was excellent for fluid T2 hyperintensity (κ = 0.88 [95% CI: 0.81; 0.94]) and substantial for T2 signal inversion (κ = 0.74 [95% CI: 0.65; 0.84]). Overall reproducibility of the WHO-IWGE stage was excellent (ICC = 0.87 [95% CI: 0.79; 0.93]). Stage-specific κ values showed the best concordance for CE1 (κ = 0.83 [95% CI: 0.66; 0.96]) and CE3 (κ = 0.82 [95% CI: 0.72; 0.91]), acceptable agreement for CE4 (κ = 0.70 [95% CI: 0.59; 0.80]) and CE2 (κ = 0.65 [95% CI: 0.28; 0.90]), and only moderate agreement for CE5 (κ = 0.57 [95% CI: 0.38; 0.75]). Anatomical location influenced the overall WHO stage reliability: hepatic cysts showed substantial agreement (κ = 0.75 [95% CI: 0.65; 0.83]), whereas pulmonary cysts achieved only fair agreement (κ = 0.42 [95% CI: 0.11; 0.74]).

### Matrix imaging features according to the WHO-IWGE classification

The MRI and CT characteristics of the cyst matrix varied significantly with the WHO-IWGE stage (Tables 2, 3). A fatty T1 hypersignal was significantly more common in inactive cysts (20/97; 21%) compared to active/transitional cysts (2/77; 3%) (*p* < 0.001). This feature was particularly prevalent in the CE4 stage, where it was observed in 18/75 cysts (24%). A non-fatty T1 hypersignal was infrequent and showed no significant difference between the inactive group (7/97; 7%) and the active/transitional group (3/77; 4%) (*p* = 0.344).

**Table 2:** CT and MRI features associated with the WHO-IWGE classification

	CE1	CE2	CE3a	CE3b	CE4	CE5	
	*N* = 17 (10)	*N* = 10 (6)	*N* = 12 (7)	*N* = 38 (22)	*N* = 75 (43)	*N* = 22 (13)	*p*-value
T1-weighted imaging							
Fatty hypersignal	0 (0)	0 (0)	0 (0)	2 (5)	18 (24)	2 (9)	0.006
Non-fatty hypersignal	0 (0)	0 (0)	0 (0)	3 (8)	6 (8)	1 (4.5)	0.69
Isosignal	1 (6)	2 (20)	5 (42)	38 (100)	69 (92)	19 (86)	< 0.001
Hyposignal	17 (100)	10 (100)	12 (100)	36 (95)	73 (97)	22 (100)	0.705
T2-weighted imaging							
Fluid hypersignal	17 (100)	10 (100)	12 (100)	38 (100)	0 (0)	0 (0)	< 0.001
Non-fluid hypersignal	0 (0)	0 (0)	2 (20)	37 (97)	74 (99)	16 (72)	< 0.001
Isosignal	0 (0)	0 (0)	1 (8)	36 (95)	70 (93)	19 (86)	< 0.001
Hyposignal	0 (0)	0 (0)	1 (12)	35 (92)	65 (87)	21 (95)	< 0.001
Matrix flip-flop sign	0 (0)	0 (0)	0 (0)	2 (5)	51 (68)	10 (45)	< 0.001
Calcifications							
External membrane calcifications	1 (6)	4 (40)	3 (25)	20 (53)	48 (64)	18 (81)	< 0.001
Endocyst membranes calcifications	0 (0)	1 (10)	1 (8)	0 (0)	12 (16)	10 (45)	< 0.001
Matrix calcifications	0 (0)	0 (0)	0 (0)	2 (5)	28 (37)	18 (81)	< 0.001

Numbers in parentheses are percentages

**Table 3:** Comparison of cysts’ imaging features according to their location, before and after adjustment on the WHO-IWGE classification

WHO-IWGE	Extrahepatic cyst location *N* = 29	Hepatic cyst location *N* = 145	*p*-value	Adjusted *p*-value
classification			0.024	
CE1	6 (21)	11 (8)		
CE2	1 (3)	9 (6)		
CE3a	3 (10)	9 (6)		
CE3b	10 (34)	28 (19)		
CE4	6 (21)	69 (47)		
CE5	3 (10)	19 (13)		
Maximal size (cm)	6.19 ± 4.63	5.74 ± 3.86	0.582	0.266
T1-weighted imaging				
Fatty hypersignal	0 (0.0)	22 (15)	0.053	0.143
Non-fatty hypersignal	1 (3)	9 (6)	0.884	0.597
Hyposignal	25 (86)	145 (100)	**< 0.001**	0.849
T2-weighted imaging				
Fluid hypersignal	20 (69)	57 (39)	**0.004**	0.068
Non-fluid hypersignal	21 (72)	108 (75)	1.000	**0.017**
Isosignal	15 (52)	111 (77)	**0.012**	0.818
Hyposignal	16 (55)	106 (73)	0.074	0.231
Daughter cyst (number)	0 (0; 4)	0 (0; 0)	0.151	0.784
Daughter cyst size	1 (0.95, 1.6)	3 (2; 4.3)	**0.008**	**0.039**
Internal detached membranes	6 (21)	74 (51)	**0.005**	0.093
Matrix flip-flop sign	5 (17)	58 (40)	**0.021**	0.427
Outgrowth	7 (24)	27 (19)	0.669	0.668
Fissuration	9 (31)	10 (7)	**0.001**	**0.002**
Calcifications				
External membrane	8 (28)	86 (59)	**0.003**	**0.020**
Internal membranes	3 (10)	21 (15)	0.768	0.831
Cyst matrix	4 (14)	44 (30)	0.111	0.485

Numbers in parentheses are percentages

Numbers in parentheses are median and interquartile range

Bold values indicate statistically significant results, corresponding to *p* values below the predefined significance level (*p* 0.05)

On T2-weighted imaging, a fluid hypersignal was a universal finding in all active and transitional cysts (77/77; 100%; stages CE1 through CE3b) and completely absent in inactive cysts (0/97; 0%) (*p* < 0.001). A notable finding was the MRI matrix flip-flop sign, which was predominantly observed in inactive cysts (61/97; 63%), particularly in stages CE4 (51/75; 68%) and CE5 (10/22; 45%). This was never found in active cysts; it was only seen in transitional cysts: 2/77 cases (3%), both of the CE3b stage (*p* < 0.001).

Calcifications demonstrated a clear progression across the stages of cyst evolution. Cyst external membrane calcifications were significantly more frequent in inactive cysts (66/97; 68%) than in active/transitional cysts (28/77; 36%) (*p* < 0.001), more precisely 5/27 (19%) in the active group and 23/50 (46%) in the transitional group. Similarly, both parasitic membranes calcifications (22/97; 23% vs 1/77; 1%) and matrix calcifications (46/97; 47% vs 2/77; 3%) were significantly associated with the inactive stages (both *p* < 0.001).

Diagnostic performance metrics were then calculated for the main ancillary signs to identify inactive cysts (CE4–CE5) versus active/transitional cysts (CE1–CE3b). A fatty T1 hypersignal showed a low sensitivity (20/97; 20.6%, 95% CI: 13.1–30.0) but a high specificity (75/77; 97.4%, 95% CI: 90.9–99.7). Its positive predictive value (PPV) was 90.9% (20/22; 95% CI: 70.8–98.9), and the negative predictive value (NPV) was 49.3% (75/152; 95% CI: 41.1–57.6). The MRI matrix flip-flop sign demonstrated a higher sensitivity (61/97; 62.9%, 95% CI: 52.5–72.5) while maintaining a high specificity (75/77; 97.4%, 95% CI: 90.9–99.7), yielding a PPV of 96.8% (61/63; 95% CI: 89.0–99.6) and an NPV of 67.6% (75/111; 95% CI: 58.0–76.1). Finally, matrix calcifications provided an intermediate sensitivity (46/97; 47.4%, 95% CI: 37.2–57.8) with similarly high specificity (75/77; 97.4%, 95% CI: 90.9–99.7); the corresponding PPV was 95.8% (46/48; 95% CI: 85.7–99.5) and the NPV was 59.5% (75/126; 95% CI: 50.4–68.2). Overall accuracy was 54.6% for fatty T1 hypersignal, 78.2% for the matrix flip-flop sign, and 69.5% for matrix calcifications.

### Refining inactive CE cysts classification

Among the 97 inactive CE cysts, 24 (25%) were classified as early, 61 (63%) Intermediate and 12 (12%) late. A hyperintense fatty T1 signal was detected in 20/97 lesions (20%) with no significant variation observed across the various stages (Early 4/24, 16.7% vs Intermediate 16/61, 26.2% vs Late 0/12, 0%; *p* = 0.10). The proportion of cysts exhibiting external cyst wall calcifications rose from 14/22 (64%) in the Early group to 42/52 (81%) in the Intermediate and 10/11 (91%) in the Late group (*p* = 0.14). Conversely, calcified detached endocyst was observed significantly more frequently in late CE stage (Early 2/22, 9.1% vs Intermediate 13/52, 25.0% vs Late 7/11, 63.6%; *p* = 0.003), and a similar trend was observed for the presence of internal matrix calcifications (Early 5/22, 22.7% vs Intermediate 31/52, 59.6% vs Late 10/11, 90.9%; *p* < 0.001).

### Matrix imaging features according to cyst location

The distribution of WHO-IWGE stages differed significantly between hepatic and extrahepatic locations (*p* = 0.024) (Table 3). After adjusting for the WHO-IWGE classification, several location-based differences were noted. While no significant differences were observed for T1-weighted matrix signals, daughter cyst size was significantly greater in hepatic cysts (median: 3.0 cm) compared to extrahepatic cysts (median: 1.0 cm) (adjusted *p* = 0.039). Cyst fissuration was significantly more prevalent in the extrahepatic group (9/29; 31%) than in the hepatic group (10/145; 7%) (adjusted *p* = 0.002). External membrane calcifications were significantly more common in the liver (86/145; 59%) than in extrahepatic sites (8/29; 28%) (adjusted *p* = 0.020). However, no significant difference was found for the presence of internal calcifications (parasitic membranes or matrix) between the two location groups.

### Distinctive imaging features between CE and differential diagnoses

The differential diagnosis group (*n* = 30) consisted primarily of hemorrhagic biliary cysts (16/30; 53%), epidermoid cysts (5/30; 17%), and other etiologies. A comparison of imaging features revealed several key distinctions (Table 4).

**Table 4:** Comparison of cystic echinococcosis cysts and differential diagnosis

	Cystic echinococcosis cysts	Cystic echinococcosis mimickers	
	*n* = 174	*n* = 30	*p*-value
T1-weighted imaging			
Fatty hypersignal	22 (13)	0 (0)	**0.049**
Non-fatty hypersignal	10 (6)	23 (77)	**< 0.001**
Isosignal	133 (76)	21 (70)	0.598
Hyposignal	170 (98)	19 (63)	**< 0.001**
T2-weighted imaging			
Fluid hypersignal	77 (44)	26 (87)	**< 0.001**
Non-fluid hypersignal	129 (74)	17 (57)	0.082
Isosignal	126 (72)	21 (70)	0.959
Hyposignal	122 (70)	7 (23)	**< 0.001**
CT features			
Parietal calcifications	94 (54)	16 (53)	0.680
Internal calcifications	56 (32)	2 (7)	**0.006**

Numbers in parentheses are percentages

Bold values indicate statistically significant results, corresponding to *p* values below the predefined significance level (*p* 0.05)

A fatty T1 hypersignal, present in 22/174 (13%) of CE cysts, was not observed in the differential diagnosis group (0/30; 0%) (*p* = 0.049), yielding a PPV of 100% (95% CI: 84.6–100%). Conversely, a non-fatty T1 hypersignal was significantly more characteristic of differential diagnoses (23/30; 77%) than of CE (10/174; 6%) (*p* < 0.001), yielding a negative predictive value for CE (i.e., NPV for CE) of 95.9% (164/171; 95% CI: 91.7–98.3).

No significant difference was found in the prevalence of parietal calcifications between CE cysts (94/174; 54%) and differential diagnoses (16/30; 53%) (*p* = 0.680). However, internal calcifications were strongly associated with CE, being present in 56/174 (32%) of cases, compared to only 2/30 (7%) in the differential diagnosis group (*p* = 0.006).

## Discussion

Our study evaluated the MRI and CT features of CE according to the WHO-IWGE staging system, with particular attention to matrix-related signs that may help in both activity assessment and differential diagnosis. By correlating cross-sectional imaging with targeted ultrasound in equivocal cases and longitudinal follow-up, we aimed to determine whether supplementary imaging features could overcome the well-recognized limits of fluid signal analysis alone. T2-weighted sequences—particularly those acquired with very long echo times, such as the heavily T2-weighted single-shot fast spin-echo sequences used in MRCP—are able to accentuate subtle differences in cyst-fluid composition. Accordingly, T2 signal behavior is regarded—in conjunction with ultrasound—as a reference standard for determining cyst activity [6, 7]. In our protocol, ultrasound was limited to cases in which MRI findings were equivocal and was combined with systematic imaging follow-up to establish definitive activity status.

We describe a new inactivity MRI feature—the “matrix flip-flop” sign—that provided useful evidence for inactivity CE cysts assessment. We therefore propose a new three-tier classification of inactive CE cysts based on the T2-weighted MRI appearance. As soon as intracystic fluid disappears, a faint, homogeneous T2 hyperintensity persists—defining the early-inactive stage, which signals the onset of endocyst membranes mineralization. Ongoing degeneration yields the intermediate-inactive stage, typified by the “matrix flip-flop” pattern (alternating low- and high-T2 signal) that reflects progressive matrix mineralization. This pattern was encountered across anatomical locations and, after accounting for WHO-IWGE stage, was not independently associated with hepatic versus extrahepatic presentation, supporting a predominantly stage-driven phenomenon. Ultimately, when the cyst contents become completely solid with a uniformly low T2 signal, the lesion has reached the late-inactive stage (CE5), corresponding to dense, confluent calcification and debris agglutination. This three-step framework links pathophysiology directly to imaging, offering a potential objective roadmap for longitudinal monitoring and therapeutic assessment of inactive CE cysts. Thus, follow-up protocols may be adapted to the inactivity stage, with closer imaging for early and intermediate lesions and markedly reduced—or even discontinued—surveillance once the late-inactive stage is documented.

The diagnostic utility of fatty foci within the matrix for staging CE remains uncertain because published data are largely confined to isolated case reports. Most describe a fat–fluid level within CE cysts that communicate widely with the biliary tree [9]. In a following correspondence, Beric et al [10] proposed that such fat deposition may simply reflect cyst degeneration rather than biliary influx. Our findings support this interpretation: small intralesional fat foci were observed almost exclusively in inactive cysts, and none of our patients had gross cysto-biliary fistulas. Like the newly introduced “matrix flip-flop” sign, intralesional fat therefore represents a highly specific marker that reinforces the diagnosis of CE. A homogeneous non-fatty T1 hyperintensity was observed in a sizeable proportion of non-parasitic cysts, yet in none of the CE cysts, reinforcing its role as a negative predictor for CE and a practical discriminator in daily practice reporting. Internal calcifications were detected in numerous inactive (CE4–CE5) lesions but were exceedingly uncommon in active cysts or in MCE, underscoring its potential utility to suspect inactivity and supporting a confident diagnosis of CE when the differential remains uncertain. In consideration of the newly described T1- and T2-based signs, the presence of calcification contributes an additional dimension of specificity to the multimodal evaluation of cystic echinococcosis.

After adjustment for WHO-IWGE stage, intrinsic T1- and T2-weighted signal characteristics did not differ between hepatic and extrahepatic lesions. The single parameter that varied significantly was wall calcification, which occurred more frequently in liver cysts, supporting the hypothesis that cyst wall calcification may be driven by the hepatic pericyst micro-environment. Staging pulmonary CE remains challenging: inter-reader agreement was lower for lung than for liver cysts (probably owing to respiratory motion, partial-volume averaging, and inherently lower soft-tissue contrast in the thorax) [14, 15]. Development of standardized lung-dedicated MRI protocols—combining ultra-short echo-time motion-robust sequences to better depict the lung cysts [16], alongside heavily T2-weighted sequences for fluid characterization—may improve pulmonary CE assessment and inform surgical planning [17].

This study had some limitations. First, not all patients underwent an ultrasound, which was only performed in case of doubtful T2-weighted imaging. Nevertheless, as every examination included at least two T2 MRI sequences, liquid T2 hypersignal could be appreciated accurately. Then, as there are variations in the T2-weighted imaging technique according to manufacturers, this could introduce some bias in our analysis, although all the MRIs were reviewed by the same readers. Finally, in our cohort, there was a small proportion of CE1 and CE2 cysts, whereas we observed a majority of CE4 and CE5 cysts.

In conclusion, although CE activity is mainly driven by the depiction of fluid content, other cyst matrix imaging features may help evaluate CE cysts. Careful evaluation of the matrix on T2-weighted sequences allows deriving a new three-tier classification of inactive cysts and provides a logical framework for stage-adapted “watch-and-wait” strategy follow-up. Additional matrix findings—such as an intralesional fatty focus, non-fatty T1 hyperintensity, or matrix calcifications—are also useful criteria to evaluate CE activity and help differentiate CE cysts from their imaging mimickers.

## Data Availability

The datasets used and analyzed during the current study are available from the corresponding author upon reasonable request.

## Abbreviations

CE: Cystic echinococcosis
CT: Computed tomography
HU: Hounsfield unit(s)
ICC: Intraclass correlation coefficient
IQR: Interquartile range
MCE: Mimickers of cystic echinococcosis
MDT: Multidisciplinary team
MRCP: Magnetic resonance cholangiopancreatography
MRI: Magnetic resonance imaging
NRC-E: National Reference Center on Echinococcoses
US: Ultrasonography
WHO-IWGE: World Health Organization–Informal Working Group on Echinococcosis
